# Professor De Carro's Letter to Dr. Pearson

**Published:** 1801-02

**Authors:** J. De Carro

**Affiliations:** Vienna


### Profeffor De Carro's Letter to Dr. Pearson.

Dear Sir,

I CANNOT wait any longer to communicate to you my further fucceffes in the Vaccine-pock Inoculation. A dreadful Small-pox epidemic has given, this autumn, a new luftre to that practice which, according to the fenfation that it is making in this town, is not likely to be ever laid afide.

This extenfion of practice, which hitherto is entirely, or nearly fo, in my hands, has enabled me to eftimate *the accuracy of your obfervations on the inefficacy of a Cow-pock that comes too rapidly, and which does not follow the laws of that difeafe.* I muft candidly confefs to you, that till this autumn I had not a very exact idea of the Vaccine puftule; that is to fay, I knew very well how it fhould have been, to be fimilar to the defcription of the Englifh Inoculators, fuch as I had feen it on my own children: but I was not aware that it muft be abfolutely abfolutely fo, and that every appearance after inoculation was not to be looked upon as a true Vaccina. The aftonifhing likenefs of my Vaccine puftules in my fixty laft inoculations, has given me a true knowledge of it; and I fcarcely believe that I could be in future miftaken. On the third or fourth day one perceives a flight elevation and rednefs; it increafes in a veficular form till the twelfth or thirteenth day, and contains always the moft limpid fluid; a fever comes on the eighth or ninth day, or fometimes is not perceptible; the beautiful areola appears on the feventh day, and increafes till the cruft is entirely formed; this cruft begins, commonly, on the twelfth, and becomes quite black.

I have had fome cafes originating from Count Mottet's matter, where a patient has received the Small-pox by inoculation, three by natural contagion, and one has retaken the Vaccina in its perfect form. Luckily for the fuccefs of the inoculation, the parents of thefe children were advifed previoufly of the idea I had of the imperfection of their former Vaccina, and they were not aftonifhed at it, after having had a great difficulty in believing that the fuperficial cruft it had formed would not be fufficient to preferve them from future infection.

I have had two cafes where the children were already infected with the Small-pox at the time of the Vaccine inoculation, and where it fhewed itfelf as ufually, without interrupting, in any degree, the courfe of the Cow-pock. One of them proved exceffively mild, the other is ftill dubious at the time I am writing.

I have had a Cafe where the inoculated fpot gave figns of inflammation only on the twelfth day, and where the puftule has been perfectly regular and complete. It was the fame fubject that had been, feven months ago, inoculated wlth the imperfect Cow-pock.

I have not yet feen any puftules, either of the *fugitive* or of the *variolous-like* appearance, although, according to Doctor Woodville's obfervation, they fhould appear more commonly where the Small-pox is epidemic: There never was, perhaps, fuch a difaftrous epidemic at Vienna, as that we have now. It is certainly owing to it that people think fo much now of the Cow-pock, againft which they have been exceedingly incredulous. I hope to have it in my power to continue it all the winter.

By the laft accounts I have of the progreffes of the Vaccine Inoculation at Geneva, they are exceedingly rapid. They have lately obferved fome eruptive Cafes; but I need not enter into thefe particulars, as you will have them immediately from Drs. Pechier and Odier, or from the *Bibliotheque Britannique.*

I have

I have had, lately, two opportunities to make experiments upon perfons who have *undoubtedly* had the Small-pox in their infancy; they are both medical men, and confequently their teftimony is more admiffible than that of moft men. *Upon one, the Vaccine matter produced no effect whatever; and upon the other, a very small, superficial vesicle appeared, which soon changed into a crust.* In one word, there was not the leaft appearance of the true and ufual Cow-pox puftule. *They confirm your principle, that one cannot have the Cow-pock after the Small-pox.*

One fingle circumftance vexes me in my Vaccine practice: It is the frequency of inoculations that fail; that is to fay, where no puftule nor inflammation is produced. I can fcarcely attribute it to my method, which is the common puncture of moft practitioners. I make it as fuperficial as I can, and avoid as much as poffible to draw blood, which, I believe, can wafh off the matter. I make one puncture on each arm.

I fee that, in England as well as abroad, Inoculators are divided upon the advantages of the puncture or of the incifion: You and Dr. Jenner feem to make ufe of the puncture; Dr. Woodville recommends ftrongly the incifion. At Geneva they prefer the incifion; and here, the puncture. I propofe now to make comparative trials, and inoculate my firft twenty patients at one arm, with the puncture; at the other with the incifion. The difficulty of making the moft fuperficial incifion, without drawing blood, has made me hitherto adverfe to that method. Upon the whole, I diflike much to be fo often obliged to repeat the inoculation. You will oblige me very much to enter with me into fome particulars upon this point.

My fecond patient, where the two difeafes have gone on together, has had alfo a very favorable Small-pox. Do fimilar Cafes in England authorize you to believe that the Cow-pock can render the Small-pox milder, when they attack the fame fubject together?

The medical world is waiting with impatience for the Work you have promifed in your *Statement, &c.* I join my intreaties to thofe that muft already have been addreffed to you, upon the publication of a Work which muft throw the greateft light on a fubject that engages now the attention of all civilized nations. Whenever my materials fhall be fufficient, I propofe writing a complete Treatife on the fubject, for the ufe of the Continental Phyficians, and fuch perfons who are not in the way of collecting from the various torrent of information, which a little zeal and activity have opened to me. I have the honor to be, DEAR SIR,

With the fincereft efteem, &c.

*Vienna, Oct.* 22, 1800. J. DE CARRO.

P. S. I fent lately fome Vaccine matter to Lord Elgin, at Conftantinople, for the inoculation of his only fon.

